# Facing challenges with hope: universal immune cells for hematologic malignancies

**DOI:** 10.20892/j.issn.2095-3941.2022.0759

**Published:** 2023-05-04

**Authors:** Yuqing Wang, Ruihao Huang, Zheng Wang, Jingkang Xiong, Xiaoqi Wang, Xi Zhang

**Affiliations:** 1 Medical Center of Hematology, Xinqiao Hospital, State Key Laboratory of Trauma, Burn and Combined Injury, Army Medical University, Chongqing 400037, China; 2 Jinfeng Laboratory, Chongqing 400037, China

**Keywords:** Universal immune cells, graft-versus-host disease, immune tolerance, chimeric antigen receptor

## Abstract

Many patients have achieved a favorable overall survival rate since allogenic hematopoietic stem cell transplantation (allo-HSCT) has been widely implemented to treat hematologic malignancies. However, graft-versus-host disease (GVHD) and complications of immunosuppressive drugs after allo-HSCT are the main causes of non-relapse mortality and a poor quality of life. In addition, GVHD and infusion-induced toxicity still occur with donor lymphocyte infusions (DLIs) and chimeric antigen receptor (CAR) T-cell therapy. Because of the special immune tolerance characteristics and anti-tumor ability of universal immune cells, universal immune cell therapy may strongly reduce GVHD, while simultaneously reducing tumor burden. Nevertheless, widespread application of universal immune cell therapy is mainly restricted by poor expansion and persistence efficacy. Many strategies have been applied to improve universal immune cell proliferation and persistence efficacy, including the use of universal cell lines, signaling regulation and CAR technology. In this review we have summarized current advances in universal immune cell therapy for hematologic malignancies with a discussion of future perspectives.

## Introduction

Hematopoietic stem cell transplantation (HSCT) has provided hope for patients with hematologic malignancies since 1957^1^. Indeed, HSCT maintains the final therapy status for most intractable hematologic malignancies. Implanted hematopoietic stem cells reconstruct the host’s immune system through development and differentiation, and generate effector killer cells that target leukemia cells. Allogeneic HSCT was the first cell therapy applied for treating hematologic malignancies; however, due to the lack of knowledge involving human leukocyte antigen (HLA) matching, the first trial failed. The protocol revolution, the maturation of haploid-identical allogenic (allo)-HSCT, and the establishment of a registry for umbilical cord blood stem cells have made allo-HSCT possible for many patients. Currently, allo-HSCT is the most effective and widely recognized cell therapy for hematologic malignancies. Owing to severe graft-versus-host disease (GVHD) and complications from immunosuppressive drugs, patients have significantly compromised quality of life after allo-HSCT, and relapse after allo-HSCT remains the major cause for treatment failure. Indeed, approximately 40% of patients relapse after allo-HSCT^2^.

GVHD poses a major challenge for patients undergoing allo-HSCT. Chimeric antigen receptor (CAR)-T cells have a major role in hematologic malignancies. With the design of different CAR structures, applications of CAR-T therapies have been expanded from B-cell to other hematologic malignancies^3^; however, cytokine release syndrome (CRS) in patients undergoing autologous CAR-T-cell therapy and GVHD to allogenic CAR-T cells limit for further application^4,5^.

After allo-HSCT, donor-derived effector T cells have the capacity to induce both graft-versus-leukemia (GVL) and GVHD. Allogenic T cells recognize residual tumor cells, possibly *via* tumor-specific antigens, and induce apoptosis of tumor cells to reduce the risk of relapse, which is referred to as the GVL effect^6^. In contrast, the graft directly activates host antigen presenting cells (APCs) because of mismatched HLAs. Donor T cells are stimulated by APCs to act as effectors and target normal tissues, resulting in GVHD^7^. The high cytotoxicity of effector T cells leads to simultaneous rejection of residual tumors and normal tissue cells, but the immunosuppressive agents used for GVHD treatment increase relapse risk. The key problem of current cell therapies is that GVL and GVHD are both influenced by traditional immune suppression, and therefore both a low relapse rate and low incidence of GVHD cannot be achieved.

The advent of universal immune cells brings the hope to integrate high GVL and low GVHD. Universal immune cells are a composite of cells that are capable of evading immune surveillance^8^, and include intrinsic immune cells [natural killer (NK) cells, virus-specific T (VST) cells, NKT cells, yδ T cells, and macrophages], and edited universal cells. Universal immune cells can distinguish tumor cells from both donor- and host-derived normal cells and evade immune detection of the host without being attacked by the immune system of the recipient (transplantation tolerance). All universal immune cells are stimulated in a major histocompatibility complex (MHC)-independent manner. When universal immune cells are exposed to tumor cells, universal immune cells become activated for tumor rejection and do not target normal cells regardless of derivation.

As allogenic cells, universal immune cells are rarely rejected by the host immune system and always lead to transplantation tolerance; thus, universal immune cells are possible candidates for allogenic transplantation. This ability is superior to the ‘typical’ immune tolerance (self-tolerance), which allows intrinsic cells to be distinguished from extrinsic cells. Such special tolerance ability confers universal immune cells with the capacity to avoid recognition of host APCs, with reduced potential for GVHD.

**Figure 1:**
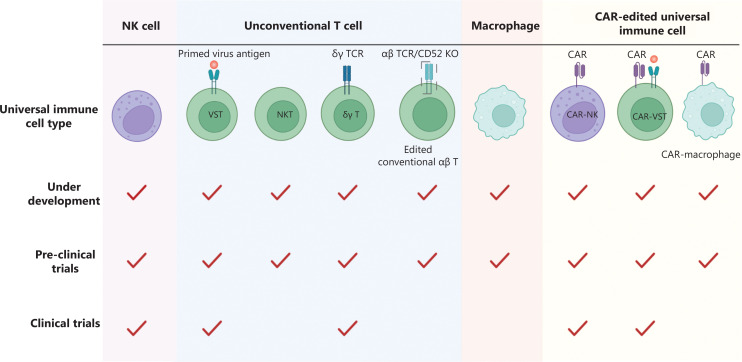
Development phases of universal immune cell therapy in hematologic malignancies. The development process of each universal immune cell therapy in hematological malignancies is divided into three phases (under development, pre-clinical trials, and clinical trials). All types of universal immune cells have been proved to maintain immuno-tolerance and have the ability to target tumor cells *in vitro*, as marked by phase 1: under development. Phase 2 (pre-clinical trials) indicates that the efficacy of universal immune cells has been tested *in vivo*. The last stage to achieve universal immune cell therapy is clinical trials. The red ticks in the figure denote that the development of specific universal immune cells has reached the indicated phase. NK, natural killer; VST, virus-specific T; NKT, natural killer T; TCR, T-cell receptor; KO, knockout; CAR, chimeric antigen receptor. The figure was created with BioRender (BioRender.com).

Universal immune cells are important in the field of cell therapy. Most patients tolerate VST-cell infusion after HSCT^9,10^, as well as haploidentical NK cell infusion after HSCT^11^. Thus, universal immune cell infusions may cause less or even no GVHD. Overall, with a strong anti-tumor ability and low GVHD occurrence because of low immunogenicity, universal immune cell therapy may be utilized. Universal immune cells are currently being developed. Because of the diverse characteristics of universal immune cells, development phases differ (**Figure 1**). We have summarized the published clinical trials of universal immune cell therapies in **Table 1**; however, unsatisfactory proliferative and persistent efficacy of universal cells are two significant problems that hinder the rapid development of universal immune cell therapy. Various strategies have been developed to comprehensively enhance efficacy, expansion, and persistence. Herein we have reviewed the immune tolerance mechanisms underlying various universal immune cells, discussed strategies to improve efficacy, and presented clinical perspectives.

**Table 1:** Published clinical trials of universal immune cell therapies

ID	Treatment	Phase	Enrollment	Disease	Efficacy				Safety	Ref
					OS/EFS	ORR	SD	PD		
NCT00187096	KIR-HLA mismatched NK cells	I	10	AML	2-y EFS: 100%	NA	NA	NA	No CRS No GVHD	^12^
NCT00799799	KIR mismatched NK cells	I	13		NA	54% (7/13)	NA	NA	No CRS No GVHD	^13^
NCT01385423/NCT02395822	Haploidentical NK cells + rhIL-5	II	42		1-y OS: 100%	35% (14/40)	0	65% (26/40)	6 CRS	^14^
UMIN000014072	Auto-NK cells + rituximab	I	9	B cell lymphoma	NA	78% (7/9)	0	22% (2/9)	No CRS No GVHD	^15^
NCT01898793	Allogeneic memory-like NK cells	I	9	R/R AML	OS: 55%	44% (4/9)	0	44% (4/9)	No DLT No CRS No GVHD	^16^
NCT00900809	NK-92 cells	I	7		NA	0	14% (1/7)	71% (5/7)	No DLT No CRS No GVHD	^17^
NCT00058812	EBV-specific CTLs	I	114	EBV-LPD after transplant	NA	85% (11/15)	0	0	No CRS 114 aGVHD 108 cGVHD	^18^
NA	EBV-specific CTLs	I	49		NA	68% (13/19)	0	21% (4/19)	No CRS No GVHD	^19^
NCT00062868/NCT01956084	LMP1/2-specific T cells	I	26		2-y OS: 68% 2-EFS: 46%	NA	NA	NA	1 DLT No CRS 5 aGVHD 3 cGVHD	^20^
NA	iNKT cells	I	9	Advanced melanoma	NA	0	67% (6/9)	33% (3/9)	No DLT No CRS No GVHD	^21^
NA	γδ T cells	I	18	R/R NHL or MM	NA	17% (3/18)	17% (3/18)	67% (12/18)	No DLT No CRS No GVHD	^22^
NA	γδ T cells	I	4	R/R T-NHL, AML, MM	NA	75% (3/4)	0	0	No CRS No GVHD	^23^
NCT03415100	NKG2D CAR-NK cells	I	3	Colorectal cancer	NA	0	100%	0	No DLT 1 CRS 1 GVHD	^24^
NCT03056339	Anti-CD19 CAR-NK cells	I	11	R/R NHL or CLL	NA	64% (7/11)	0	0	No CRS No GVHD	^25^
NCT00840853	Anti-CD19 CAR-VST cells	I	8	B-ALL or B-CLL	NA	50% (4/8)	13% (1/8)	37% (3/8)	No DLT No CRS No GVHD	^26^
NCT03294954	Anti-GD2 CAR-NKT cells	I	3	R/R neuroblastoma	NA	33% (1/3)	67% (2/3)	0	No DLT No CRS No GVHD	^27^
NCT02808442/NCT02746952	Anti-CD19 UCAR-T cells	I	21	R/R ALL	OS: 55%	67% (14/21)	0	0	19 CRS 2 aGVHD	^28^

KIR, killer cell immunoglobulin-like receptor; HLA, human leukocyte antigen; NK, natural killer; AML, acute myeloid leukemia; OS, overall survival; EFS, event-free survival; ORR, overall response rate; SD, stable disease; PD, progressive disease; CRS, cytokine release syndrome; GVHD, graft-versus-host disease; R/R, refractory/relapsed; EBV, Epstein–Barr virus; EBV-LPD, Epstein–Barr virus-positive lymphoproliferative disease; LMP, latent membrane protein; iNKT, invariant NKT; NHL, non-Hodgkin lymphoma; MM, multiple myeloma; NKG2D, NK group 2 member D; T-NHL, T cell non-Hodgkin lymphoma; CLL, chronic lymphoblastic leukemia; CAR, chimeric antigen receptor; UCAR, universal chimeric antigen receptor; ALL, acute lymphoblastic leukemia; rhIL, recombinant human interleukin; DLT, dose limited toxicity; CTL, cytotoxic T lymphocyte; aGVHD, acute graft-versus-host disease; cGVHD, chronic graft-versus-host disease.

## Immune tolerance – special immune surveillance mechanisms

### Natural killer cells

As one of the key components of the innate immune system, NK cells quickly respond to the presence of defective cells without the antigen-presenting process and actively lyse tumor or infected cells. The fate of NK cells is determined by integration of stimulatory and inhibitory signals from the immune microenvironment rather than relying on the antigen-presenting process. The ‘missing-self’ model is used to illustrate the fate-determining mechanisms underlying NK cells^29^. MHC-binding killer cell immunoglobulin-like receptors (KIRs) of NK cells bind various MHC class I molecules on healthy cells to sustain a silent NK cell state. In some tumor cells MHC class I molecules are downregulated on cell surfaces to evade effector T cell cytotoxicity, decreasing inhibitory signals in the immune microenvironment. A balance is induced to skew stimulatory signals by tumors so that the advantageous stimulatory signals driving NK cells are activated and respond to tumors^30^. Thus, NK cells recognize normal and tumor cells when MHC molecules are mismatched, rendering NK cells a possible source of universal immune cell therapies.

After introducing allo-NK cells into a host, the mismatched KIR epitopes between the host and donor may break the balance between stimulatory and inhibitory signals and unexpectedly activate infused NK cells to cause GVHD; however, the occurrence of GVHD is much less than estimated^31^. In one study, all children with acute myeloid leukemia (AML) treated with KIR-mismatched NK cells remained in remission without GVHD for at least 3 years post-infusion^12^. A subsequent study showed that alloreactive NK cells contribute to suppression of GVHD development rather than inducing GVHD development^32^ due to secreted depression factors, such as TGF-β^33,34^. The high frequency of NK cell-induced lysis and the absence of GVHD indicate that alloreactive NK cells have potential as universal immune cells. Similar immune tolerance characteristics have been observed in NK cell lines. Among 15 patients with treatment-resistant malignancies (13 with solid tumors and 2 with leukemia or a lymphoma), all tolerated NK-92-cell-line infusion^17^. Moreover, no dose-limiting toxicity was observed in 7 refractory/relapsed (R/R) AML patients treated with activated NK-92 cell lines (3 treated with 1 × 10^9^ cells/m^2^ and 4 treated with 3 × 10^9^ cells/m^2^)^35^. The low GVHD of allo-NK-cell therapy and the safety of an allo-NK-cell infusion demonstrated that NK cells are powerful universal killers.

Specifically, adaptive NK cells comprise a subset of NK cells marked by NK group 2 member C (NKG2C), which are induced by cytomegalovirus (CMV) infection and exhibit a memory-like phenotype^36,37^. The higher quantum and better expansion ability of NKG2C^+^ NK cells in the grafts following haplo-identical transplantation and donor lymphocyte infusions (DLIs) are significantly associated with a lower risk of disease progression without compromising GVL, which demonstrated that NKG2C^+^ NK cells have the potential to dissociate GVL and GVH effects^38^. As reported, CMV-seronegative patients who underwent HSCT with CMV-seropositive adult unrelated adult donors (URDs) or sibling fully HLA-matched donors showed a much higher proportion of NKG2C^+^ NK cells than patients who underwent HSCT with CMV-seronegative donors^36^. In the same clinical trial, NKG2C^+^ NK cells became highly expanded [23% ± 5% in peripheral blood mononuclear cells (PBMCs)] and produced significantly more IFN-γ in CMV-reactive recipients at 3 months after HSCT, but NKG2C^+^ NK cells comprised only 6% of PBMCs in patients without CMV reactivity at 1 year after HSCT. These results show that NKG2C^+^ NK cells have a high expansion ability and cytotoxicity in response to CMV. Moreover, after CMV reactivity, cytotoxic NKG2C^+^ NK cells have been detected at 1 year post-HSCT, even without continuous CMV stimulation^37,39^. The functional long-term characteristics make NKG2C^+^ NK cells good candidates for universal immune cell therapy. *In vitro*-stimulated NKG2C^+^ NK cells exhibit high cytotoxicity efficiency against HLA-C-mismatched primary ALL, AML, and myelodysplastic syndrome (MDS) blasts *ex vivo*^40–42^, demonstrating the strong alloreactivity of NKG2C^+^ NK cells. Superior to conventional NK cells, NKG2C^+^ NK cells are intrinsically resistant to regulatory T (Treg) cell suppression; thus, NKG2C^+^ NK cells in the tumor microenvironment (TME) are able to maintain strong cytotoxicity. Despite no completed clinical trials involving NKG2C^+^ NK cell therapies, these cells are expected to serve as efficient universal immune cells for treating hematologic malignancies.

### Unconventional T cells

#### VST cells

Before infusion, VST cells are stimulated to proliferate and differentiate into virus-specific effectors. When re-exposed to viral antigens *in vivo*, VST cells rapidly become reactivated and target infectious cells. VST cell therapy was initially applied to treat viral infections and reactivation after HSCT. Eighty-five percent (11/13) of patients with proven or probable Epstein–Barr virus (EBV)-positive lymphoproliferative disease (EBV-LPD) achieve complete remission after EBV-specific T-cell infusion; no patients have been shown to develop *de novo* GVHD^18^. Hence, VST cell therapy is highly effective and safe for preventing and treating viral infection. Moreover, although the response rate of patients in the EBV-VST cell therapy group is equivalent to patients in the DLI group, the VST cell therapy group had a higher complete remission rate (68% *vs*. 57%) and a much lower acute (a)GVHD incident rate (0% *vs*. 17%)^19^. Bao et al.^43^ successfully stimulated donor-derived VST cells with CMV peptides and infused the CMV-VST cells into patients with persistent CMV infection after HSCT; no infusion-induced GVHD was observed. CMV-infected patients who received donor-derived CMV-VST cells did not have an increased occurrence of GVHD but did have less potential for re-treatment with anti-CMV pharmacotherapies^44^. Therefore, VST cells may have immune tolerance characteristics and serve as a source for universal immune cell therapy. Nevertheless, the mechanisms by which VST cells recognize virus antigens and quickly develop into effectors have not been established.

Among patients receiving VST cell infusions, 44% were treated with donor-derived VST cells and 19% with third-party VST cells^45^. Although donor-derived VST cell therapy has high efficacy in inhibiting viral reactions and reconstructing antiviral immunity, restrictive sources, intensive labor and long-term procedures are barriers to widespread application. To overcome these barriers, third-party VST cells have been selected to treat severe infections after HSCT^46^. No GVHD associated with VST cell infusions was observed, suggesting the high safety of third-party VST cell therapy. Moreover, a third-party VST-cell bank with 32 virus-specific lines was built by several transplantation centers for treatment of EBV, CMV, and adenovirus (AdV) infections after HSCT^47^. Seventy-four percent of patients achieved complete or partial remission 6 weeks post-infusion, and only 2 of 50 patients developed *de novo* GVHD. Tzannou et al.^9^ successfully constructed a VST cell bank recognizing five viral pathogens [EBV, AdV, CMV, BK virus (BKV) and human herpesvirus (HHV)-6]. The overall cumulative complete or partial response rate after a single infusion was 92% and 100% for BKV and EBV, respectively. For both virus infections, patients who received two types of VST cells had clinical improvement. Among 38 patients receiving VST cells, only 2 had *de novo* GVHD, which was controlled by corticosteroids^9^. Moreover, patients with B-cell EBV-associated lymphomas achieved a 2-year overall survival of 80% after VST cell therapy, strongly increasing the published post-transplantation 2-year overall survival rate of 30%^20^. These results showed that the construction of third-party VST-cell banks accelerate the production process and guarantee timely treatment of an infection, constituting an efficient strategy to treat severe infections after HSCT.

Multi-VST cells have enabled treating multiple infections through a single infusion and reducing infusion times and costs^48^. EBV-, CMV- and AdV- trispecific T cells were infused into 10 recipients with single or multiple infections after HSCT^49^. All of the patients achieved a complete response to VST-cell therapy, with the absence of immediate or delayed infusion-related toxicity. Papadopoulou et al.^50^ generated a single donor-derived VST cell culture targeting 12 antigens from 5 viruses (AdV, EBV, CMV, BKV, and HHV-6) and infused the culture into 11 patients as prophylaxis or treatment for virus infections after HSCT^50^. Ninety-four percent of the recipients achieved partial or complete response, and *de novo* GVHD was observed in only one patient, confirming the feasibility of multi-VST cells to prevent viral infection after HSCT. Future work should involve building broad-spectrum viral banks and producing integrated VST cell cultures specific for multiple viruses. This effort will contribute to large-scale production and rapid infection prophylaxis and treatment; however, the efficacy of VST-cell therapy is restricted in virus-dependent diseases, with limited expansion ability in virus-independent diseases.

#### NKT cells

NKT cells are considered as a specific type of αβ T cell, accounting for < 1% of T cells in the peripheral blood (PB)^51^. NKT cells develop in the thymus and mature to express CD3 through the same selection as conventional T cells. In contrast to conventional T cells, NKT cells possess characteristics of NK cells, including expression of NK cell markers (CD16 and CD56) and secretion of granzyme and perforin. As a bridge between the innate and adaptive immune systems, NKT cells have various roles, including direct cytolysis, cytokine secretion, and immune regulation. NKT cells are divided into two subtypes based on the diversity of the T-cell receptor (TCR) α chain [invariant NKT (iNKT) and variant NKT cells]. iNKT cells are the major subtype used for cell therapy and are discussed in detail herein.

iNKT cells express a single invariant antigen receptor to recognize the α glycolipid ligand [α-galactosylceramide (α-GalCer)] presented by CD1d in professional APCs^52^. The molecule CD1d is similar to MHC class-I molecules, but monomorphic in humans; thus, CD1d overcomes MHC incompatibility^53^. Donor iNKT cells have been infused into post-HSCT mice and were shown to infiltrate GVHD-targeted tissues, but did not cause GVHD^54^. As the infusion dose of donor iNKT cells increased, GVHD burden decreased; simultaneously. At the same time, tumor clearance by conventional T cells was not affected^54^. Moreover, iNKT cells promote proliferation of regulatory T cells, which are mainly responsible for immune suppression^55^. Thus, iNKT cells inhibit GVHDs experimentally and simultaneously maintain GVL effects. These results show that iNKT cells may be a good candidate as a source for universal immune cell therapy. Clinically, a low post-transplantation iNKT:T ratio and iNKT cell dose were both shown to be independent risk factors associated with aGVHD^56,57^. As the ratio increases, the potential for aGVHD occurrence deceases^56^. iNKT cells have a pivotal role in dissociating GVL effects and GVHD^58^. Because of the integration of special tolerance mechanisms and tumor lysis ability, NKT cells have the potential to be good tumor killers. Low-grade (grade 1 or 2) GVHD has been observed in patients with metastatic melanoma receiving iNKT infusions^21^. Although two neuroblastoma patients maintained stable disease after anti-GD2 CAR-NKT cell infusions, all three recipients tolerated the treatment well, without CRS or neurotoxicity^27^. These results demonstrated that highly immune tolerant NKT cells may lack strong efficacy in tumor killing.

Most ongoing clinical trials on NKT cell therapy involve solid tumor treatment^59^, but no completed clinical trials have been reported. The very small amounts of NKT cells, approximately 1% in the liver and 0.008%–1.176% of cells in PB^59^, make it difficult to obtain sufficient circulating NKT cells. Although tissue-specific NKT cells have been reported to be critical in GVHD inhibition^60^, the roles of circulating NKT cells are unclear, suggesting that the local immune microenvironment may be critical for NKT cells to function and the actual tumor-damage ability of circulating NKT cells may be small. The effects of NKT cells in solid tumors are possibly much better than the effects in hematologic malignancies. Because CD1d is expressed in acute lymphoblastic leukemia (ALL), AML, B-cell chronic lymphoblastic leukemia (CLL), juvenile myelomonocytic leukemia, and non-Hodgkin lymphoma (NHL)^61^, NKT cell therapy may be applied to treat CD1d-expressing hematologic malignancies.

#### yδ T cells

yδ T cells account for 1%–5% of circulating T cells^59^ and are mainly responsible for innate immune responses. yδ T cells are located in non-lymphocyte tissues and epithelial surfaces, such as the intestine and skin. yδ T cells are mainly involved in inflammation, autoimmunity, memory cell generation, and damaged tissue healing^62,63^.

There are two main mechanisms for underlying yδ T cell activation. The TCR-dependent mechanism involves yδ TCRs binding to non-peptide prenyl-pyrophosphate metabolites of isoprenoid biosynthesis^64^ or CD277^65^, which are not restricted by recognition of MHC class-I molecules. Another mechanism involves binding to MHC class I-related chain A/B (MICA/B), UL16 binding protein (ULBP), and polyoma virus receptor (PVR) on tumor cells through DNAX accessory molecule 1 (DNAM1) and natural killer cell receptors (NKRs), NKG2D, NKp30 and NKp44 on yδ T-cell membranes^66,67^. Thus, the fate of yδ T cells depends on the network of receptor-ligand interactions rather than TCR-MHC stimulation. The mechanism reduces the possibility of MHC compatibility-induced GVHD. Among 9 patients with relapsed/refractory low-grade NHL or multiple myeloma (MM), significant *in vivo* activation and proliferation of yδ T cells were observed in 55% (5/9) of the patients after yδ T-cell infusions^22^. An objective response was achieved in 33% (3/9) of patients, prompting the possible anti-tumor efficacy of yδ T cells. None of the six patients with MM had serious treatment-related adverse events after zoledronate-activated Vy9 yδ T-cell infusions^68^. Moreover, no signs of aGVHD or chronic GVHD were observed among patients with advanced refractory hematologic malignancies [one each with T cell NHL (T-NHL), AML, and secondary plasma cell leukemia, and one with MM]^23^. These results confirm that yδ T-cell therapy is highly safe; however, the unstable phenotype and poor expansion of yδ T cells pose problems for large-scale production and wide application. Clinical trials concentrating on yδ T-cell therapy for treating hematologic malignancies are ongoing to test the efficacy and explore good manufacturing practice (GMP).

#### Edited conventional αβ T cells

Conventional αβ T cells are activated in an MHC class molecule- and TCRαβ-dependent manner. Immune responses are stimulated by MHC mismatch between a donor and host, thus causing donor T cell-induced damage to normal tissues. MHC class II molecules have been confirmed to be associated with GVHDs^69,70^. No aGVHD was observed after injecting PBMCs into MHC class I- and/or II-deficient mice^71^. Downregulation of MHC class II molecules may achieve tolerance and knocking out TCRαβ may effectively prevent GVHD caused by an MHC mismatch. Approximately 20% of CD3ϵ molecules can be eliminated using the zinc finger nuclease (ZFN) pair targeting the TCR α constant region (TRAC)^72^, but transcription activator-like effector nucleases (TALENs) achieved > 70% CD52 knockout (KO) with < 1% CD3 expression^73^. Indeed, KO efficacy should be continuously improved. Because of the easier design method, reduced cost, and higher targeting efficiency, clustered regularly interspaced short palindromic repeats and CRISPR-associated protein 9 (CRISPR/Cas9) technology has the potential to achieve higher TCR-KO efficiency. The HLA-B-KO inducible pluripotent stem cell (iPSC) model has been successfully established based on the CRISPR/Cas9 system^74^, 92% of TCRαβ was eliminated^75^, which is superior to TALENs^76^. Furthermore, HLA class I, class II, and TCR triple-KO T cells show similar anti-leukemia efficacy without inducing GVHD, and HLA^null^ T cells exhibit prolonged persistence^77^; however, no clinical trials have been conducted out to determines the potential of edited conventional αβ T-cell therapy, indicating a hurdle to clinical application.

### Macrophages

In addition to the above-described universal immune cells, macrophages have become increasingly popular as a part of universal immune cell therapy. Macrophages have diverse functions, including regulating development, maintaining a tissue-specific immune environment, clearing injured cells, eliminating pathogens and participating in inflammatory responses^78^. Macrophages are separated into two main types (M1 and M2 macrophages). M1 macrophages are critical in inflammatory regulation and adaptive immune-response stimulation with potential anti-tumor ability^79^. M2 macrophages [tumor-associated macrophages (TAMs)] enhance tumor progression, promote tumor metastasis, and suppress anti-tumor immunity in the TME^80^. Many strategies have been reported to combat TAMs^79,81^. In patients with aggressive and indolent NHL, a macrophage checkpoint inhibitor combined with rituximab achieved promising outcomes, with high safety^82^; however, current clinical trials have mainly concentrated on solid tumors, perhaps due to the strong roles of tissue-specific macrophages. Undoubtedly, further clinical trials focusing on hematologic malignancies are warranted.

## Strategies to improve the efficacy of universal immune cells

**Figure 2:**
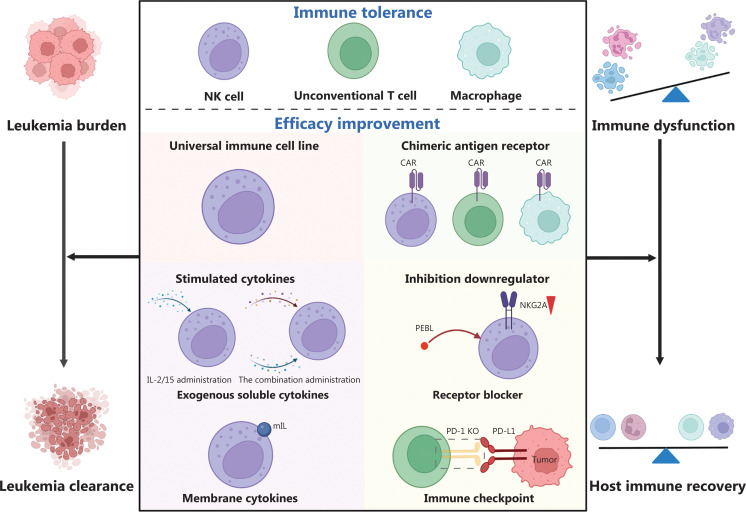
Development flow of universal immune cell therapy. On the basis of special immune tolerance, the main characteristic of universal immune cells, GVHD can be overcome. The three main types of universal immune cells are NK cells, unconventional T cells and macrophages. Through four strategies, the efficacy of universal immune cells is improved. Universal immune cell therapy may have the potential to achieve complete leukemia clearance and help the host with immune recovery. NK, natural killer; mIL, membrane IL; PEBL, protein expression blocker; NKG2A, NK group 2 member A; PD-1, programmed cell death protein 1; KO, knockout; PD-L1, programmed cell death protein 1 ligand; CAR, chimeric antigen receptor. The figure was created with BioRender (BioRender.com).

Special tolerance mechanisms of universal immune cells contribute to the low GVHD occurrence; however, poor expansion and weak persistence render this difficult. The infused NK-cell density *in vivo* peaks in adult AML patients on day 24 post-infusion, but is much less than the baseline frequency of 26 × 10^9^/L^13^. The unsatisfactory expansion efficacy of NK cells *in vivo* may cause persistent disease in 80% (4/5) of recipients. Although VST cells can achieve 13-fold expansion *in vitro*, the maximum is only 82.5 × 10^7^ cells^50^. Similarly, the yδ T-cell frequency only reaches 68-fold (4.3 × 10^7^/L) after *in vivo* expansion^23^. The total number of expanded iNKT cells *in vitro* ranges from 1.1 × 10^7^–1.26 × 10^9^, which indicates unstable proliferative efficacy^21^. Although the frequency of circulating NKT cells was shown to increase over baseline *in vivo*, the frequency rapidly decreased in 67% (2/3) of patients in week 4 post-infusion before complete tumor clearance^27^. The lack of *in vivo* CMV-specific T-cell expansion by day 21 was shown to always be associated with the absence of an anti-CMV response^83^. Moreover, CMVs were reactivated in 7 of 34 patients^9^. The insufficient durability of VST cells may be associated with loss of viral antigens. The unsatisfactory expansion and persistence of universal immune cells cause refractory problems and restrict applications, both of which need to be improved (**Figure 2**).

### NK cell line

Because NK cell activation is dependent on the signaling network in the immune environment and lacks pivotal stimulatory signals, the proliferation efficacy of infused NK cells is difficult to control, which leads to diverse clinical outcomes. Indeed, it is difficult to achieve clinical-scale production of NK cells with stable proliferation. NK cell lines may be ideal sources to fulfill standard production procedures on a large scale. Several NK cell lines from NK cell leukemias/lymphomas have been reported to have stronger proliferation efficacy, including the KHYG-1^84^, NKL^85^, YT^86^, and NK-92 cell lines^87^. Fine-quality granules form in all of cell lines, but only the KHYG-1 and NK-92 cell lines have significant cytotoxicity^88^. Moreover, the NK-92 cell line has stronger cytolytic ability and a lower IL-2 content requirement for proliferation than the KHYG-1 cell line^89^. Currently, the NK-92 cell line is one of most popular candidates for universal immune cell therapy and the sole platform exploited for clinical trials among NK cell lines. The frequency of NK-92 cells reached approximately 1 × 10^9^ cells/culture bag over 15–17 days by culturing with recombinant human interleukin-2 (rhIL-2) and 500 U/mL of proleukin^90^. Furthermore, a nearly 35-fold expansion was achieved within 216 h with 1,000 U/mL of proleukin^17^. After infusing *ex vivo*-cultured NK-92 cell lines, low toxicity to PBMCs and bone marrow hematologic cells was observed; however, possible virus positivity and the tumorgenicity of NK-92 cell lines are problematic. Although no viral particles, bacteria, fungi or mycoplasmas have been reported in the NK-92 cell lines^91^, Matsuo and Drexler^88^ detected EBV in the NK-92 cell line *via* a polymerase chain reaction (PCR) with EBV nuclear antigen (EBNA)-1 specific primers. Thus, multi-virus positivity should be further assessed and the NK-92 cell line must be evaluated for multi-virus loads before infusion. Furthermore, the NK-92 cell line must be irradiated before infusion because of tumor derivation. The genetic instability of the NK-92 cell line probably contributes to the lack of long-term antitumor efficacy, even with the maintenance of IL-7 and IL-12^87^. Thus, it is necessary to incorporate the following strategies to promote the efficacy of the NK-92 cell line.

### Stimulation cytokines

Regulatory cytokines, such as IL-2, IL-12, IL-15, IL-18, and IL-21, have significant roles in activating and maintaining universal immune cells. Early clinical trials focused on the stimulation efficacy of IL-2 on universal immune cells. The role of IL-2 in expansion enhancement and cancer drainage has been confirmed^92^. NKT cells were isolated from IL-2-cultured and α-GalCer-pulsed PBMCs. Low-dose IL-2 was also used *in vivo* to stimulate the expansion of yδ T cells^22,23,68^. Moreover, a GMP-grade protocol for NK cells has been published based on IL-2. Purified NK cells were cultured with 1,000 U/mL recombinant human (rh) IL-2 for 12 days. The NK-cell expansion rate was vigorous (30-fold) in 11.8% (2/17) of donors but varied^93^. On average, a 5-fold expansion was achieved^94^. The infusion priming content of IL-2 should be considered. A high dose of IL-2 leads to severe side effects, whereas low-dose IL-2 enhances expansion ability but has no influence on anti-tumor capacity. This finding may be caused by Treg-cell activation^95^ because Treg cells express high-affinity IL-2 receptors^96^. Thus, low-dose IL-2 potently upregulates immunosuppression and inhibits anti-tumor responses.

IL-15 is thought to be a substitute for IL-2 in stimulation of universal immune cells. IL-15 exhibits stimulatory efficacy in lymphocytes similar to that of IL-2 through the IL-15—IL-15Rα—IL-2Rβ—γc complex axis^97^. Despite different intracellular signals, immune cells cultured with IL-15 and IL-2 share highly analogous gene expression profiles^97^. IL-15 is critical for proliferation and activation of NK cells and CD8^+^ T cells, leading to stronger tumor-clearance efficacy^98,99^. Moreover, IL-15 has the capacity to trigger the NK-92 cell line without IL-2^100^. Compared to rhIL-2, rhIL-15 has a better anti-tumor effect and more significant enhancing ability on cytotoxic T and NK cells^101^, with the expansion rate and lifetime of rhIL-15-induced NK cells being significantly higher^14^. Thirty-five percent of patients with refractory AML achieved remission after treatment with a NK cell infusion and rhIL-15; however, cytokine release syndrome (CRS) and neurotoxicity occurred^14^, which may be associated with IL-15-induced prolonged drug accumulation and exposure. Compared with IL-2, systemic IL-15 promotes proliferation and activation of CD8^+^ T cells so that allo-rejection responses are accelerated^102^, demonstrating that the IL-15 infusion dose and period must be accurately controlled.

Application of cytokine panels has the potential to enhance the antitumor efficacy and expansion ability of universal immune cells. A combination of these cytokines does not contribute to a large increase in number but regulates the universal immune cell phenotype and enhances cytotoxicity^103^. IL-12, IL-15, and IL-18 together induce memory-like NK cells, leading to higher cytotoxicity when re-stimulated^16^. A 55% overall response rate and 45% complete remission (CR)/incomplete count recovery (CRi) have been achieved in relapsed/refractory AML patients with infusion of active memory-like NK cells^16^. Memory-like NK cells may be another important universal immune-cell source in the future.

Although exogenous soluble cytokines are immediately effective after infusion, exogenous soluble cytokines do not offer continuous stimulatory signals. Thus, soluble cytokines should be injected several times, with possible life-threatening side effects. Membrane cytokines have been designed to achieve long-term stimulation and reduce infusion times. Inserting IL-15 into the NK cell membrane maintains stimulation signals. NK cells with mIL-15 maintain self-survival and -expansion capacity without additional IL-2 infusions, achieving stronger antitumor ability^104^.

Over recent decades, co-culturing with feeder cell lines has been a promising method to induce activation and proliferation of universal immune cells. Feeder cells stimulate universal immune cells *via* activated cytokines and cell-cell communications. Feeder cell lines for NK cells include HFWT, K562, RPMI 1866, Daudi, KL-1, MM-170, and EBV-transformed lymphoblastoid cell lines (EBV-LCLs)^103^. Following the same strategy of feeder cells, PBMCs have been cultured with GM-CSF and IL-2 and pulsed with α-GalCer to generate APCs as NKT-cell feeder cells^105^. After co-culturing with *ex vivo*-generated APCs, a > 10-fold expansion of iNKT cells was achieved, and the increasing trend remained for at least 1 week.

Integration of feeder cells and membrane cytokines offers novel platforms to culture universal immune cells. Genetically engineered K562 cells with membrane-bound IL-15 and 41BB ligands are more effective in stimulating NK cells than IL-2, IL-12, IL-15, and/or IL-21^106^. Furthermore, K562 cells modified with mIL-21 exhibit stronger promotion efficacy against NK cells than K562 cells modified with mIL-15^107–109^. Feeder cells modified with membrane cytokines support clinical-grade expansion of highly cytotoxic universal immune cells. Further research involving the mechanisms of the interplay between cytokines and universal immune cells is worthwhile.

Upregulating activated receptors is also a good strategy. iPSC-derived NK cells have been induced to generate a point mutation of CD16a. CD16a is well known as the stimulatory receptor for NK cells and is required to maintain an active state^110^. A high-affinity non-cleavable variant of CD16a (hnCD16)-NK cells exhibits stronger antibody-dependent cell-mediated cytotoxicity (ADCC) against multiple tumor lines than PB-derived NK cells. Thus, iPSC-derived NK cells may be a source of universal immune cells.

### Downregulating inhibitory signals

In general, the fate of universal immune cells depends on the balance between active and inhibitory signals in the TME. The purpose of methods for stimulating cytokines is to upregulate active signals; downregulating inhibitory signals is another good strategy. One of the mechanisms causing the unsatisfactory anti-tumor ability of NK cells is that tissues in the TME express non-classical HLA class-I molecule HLA-E, which binds to the NK inhibitory receptor, CD94/NKG2A, and inhibits NK cells^111^. A single-chain variable fragment derived from the anti-NKG2A antibody has been linked to endoplasmic reticulum-retention domains to form NKG2A protein expression blockers (PEBLs). These PEBLs block the NKG2A transport process from the endoplasmic reticulum to the cell membrane, thus causing downregulation of inhibitory receptors on NK cells. NKG2A^null^ NK cells exhibit higher cytotoxicity and increased ADCC activity and the potential to kill tumor cells expressing HLA-E or HLA-G; however, the proliferative capacity of NKG2A^null^ NK cells may be poor in HLA-E^null^ tumor tissues because of strong inhibitory signals.

Tumors express immune checkpoint ligands to suppress immune responses in the TME to evade immune surveillance and build a tumor-friendly microenvironment, thus leading to relapse. The programmed cell death protein (PD-1)/programmed cell death 1 ligand 1 (PD-L1) pathway is an important inhibitory pathway. Immune inhibitor blockade therapy targets PD-1 to downregulate immunosuppressive roles and yields significant clinical outcomes in cancer treatment^112,113^. PD-1 knockout is associated with enhanced persistence and antitumor ability of cytokine-induced killer cells^114^. The stable tumor burden is markedly decreased after administration of the anti-PD-1 antibody to the co-culture system of exhausted mesothelin-CAR-T cells and pleural mesothelioma cells^115^, thus showing that immune inhibitor blockade therapies delay exhaustion of CAR-T cells. Compared with wild-type CAR-T cells, the density of PD-1-deficient CAR-T cells is much larger, with higher levels of IFN-γ and IL-2 in PB, which indicates that PD-1 knockout strongly prolongs survival of CAR-T cells and simultaneously enhances cytokine secretion ability^116^. Li et al.^117^ genetically-modified CAR-T cells to constitutively secrete PD-1 inhibitors. They effectively inhibited PD-1 expression on CAR-T cells and enhanced anti-tumor activity, as well as expansive efficacy. All modified CAR-T cells survived to day 80, which is much longer than non-modified CAR-T cells and the combination of anti-PD-1 antibody and non-modified CAR-T cells. Similarly, PD-1 molecules are expressed on tumor-infiltrating NK cells and suppress the anti-tumor cytotoxicity of NK cells^118^. The tumor burden was significantly decreased in the group receiving the triple combination of iPSC-derived NK cells, activated CD3^+^ T cells, and anti-PD-1 antibody compared with the group given double combination therapies^119^. The obstacle of poor expansion and weak persistence of universal immune cells may be overcome by combination anti-PD-1/PD-L1 therapy.

In addition to the PD-1 molecule, cytokine-inducible Src homology 2–containing (CIS) protein, a key inhibitor of IL-15 signaling, has been knocked out by the CRISPR/Cas9 system in CAR-NK cells to improve anti-tumor ability^120^. The modified CAR-NK cells secrete more IFN-γ and TNF-α and exhibit stronger cytotoxicity against CD19^+^ Raji lymphoma cells. Novel immune checkpoint molecules should be considered when enhancing the therapeutic efficacy of universal immune cells.

### CAR

CAR directs cytotoxic cells to concisely lyse antigen-positive tumors. After recognizing specific antigens on tumor surfaces, the CAR intracellular domain stimulates downstream signaling pathways according to the tumor burden. CAR-universal immune cells integrate the accurate target of CAR technology and the special tolerance mechanisms of universal immune cells. CAR-NK cells have been the most popular platform to explore the feasibility of CAR-universal immune cells. Notably, 63% of patients [7/11 (4 with lymphoma and 3 with CLL)] achieved complete remission with high safety^25^. At the 27th European Hematology Association (EHA) Congress, Zhang et al. reported that eighty percent (4/5) of R/R AML patients treated CD33 CAR-NK-cell therapy have achieved CR with minimal residual disease (MRD) negativity.

Currently, clinical trials concentrating on CD19-, CD22-, CD7-, and CD30-CARs are ongoing^121^. Novel CAR-target sites should include tumor antigens, and activate receptors and immune checkpoint blockade. The CAR construct was designed as NKG2D-DAP10-CD3ζ^122^ because the NKG2D-DAP complex is critical in NK-cell activation^123^. After activation by the K562-mbIL-15–4-1BBL cell line, NKG2D-DAP10-CD3ζ NK cells were re-invigorated and showed high cytotoxicity against ALL cell lines. The engineered CAR-NK cells mitigated clinical symptoms and reduced tumor burden in metastatic cancer sites^24^; however, the clinical efficacy of NKG2D-DAP10-CD3ζ NK cells on hematologic malignancies needs to be determined. As both a tumor neoantigen and an immune checkpoint blockade, HLA-G was introduced into the CAR vector^124^. Anti-HLA-G-CAR-NK cells effectively destroyed several solid tumor lines and re-stimulated Syk/Zap70, which was significantly downregulated in the immunosuppressive microenvironment. Thus, the microenvironment-regulating role of CARs should be considered when selecting neoantigens.

The ectodomain of CAR is determined by the tumor of interest, and the endo-domain depends on the signaling pathway network in universal immune cells. CAR:4-1BB-NK cells killed 77.7% of MM cells *in vitro* and exhibited enhanced cytotoxicity compared to wild-type NK cells^125^. Although CD123-CAR-NK cells with 4-1BB or 2B4 both showed significant cytotoxic efficacy against the CD123-positive AML cell line, 2B4 CAR-NK cells exhibited a long-term survival advantage^126^. After co-culturing with feeder cells, there was a dramatic increase in expression of NK-cell active markers (CD69, HLA-DR, and NKG2D) on 2B4 CD5-CAR-NK cells, whereas 4-1BB CD5-CAR-NK cells only showed a slight increase^127^. 2B4 has stronger stimulatory effects on NK cells and is superior to the intercellular domain of CAR. Moreover, the novel molecule DAP12 is a candidate intercellular molecule for invigorating CAR-NK cells. Although DAP12 CAR-YT cells have similar cytotoxicity to CD3ζ CAR-YT cells at an E:T of 10:1, DAP12 CAR-YT cells exhibit stronger anti-tumor ability at lower ratios (1:2.5 and 1:5)^128^, demonstrating the slight advantage of DAP12 with regard to NK cell stimulation.

In addition, VST cells can serve as a platform or CAR technology. Six patients who experienced relapse after HSCT were infused with CD19-CAR-VST cells, and all of the patients tolerated the allogenic cell infusions well, showing tolerance of CAR-VST-T cells^26^. In patients with viral reactivation, re-expansion of CAR-VST cells was observed simultaneously with increasing EBV loads in PB; however, a median survival time of 8 weeks revealed the poor persistence of CAR-VST cells. This unsatisfactory persistence should be improved, which may be overcome by continuous viral stimuli, such as planned vaccinations^129,130^. Moreover, iPSC-derived CAR-macrophage-cell therapy exerts good phagocytosis activity in the K562 leukemia cell line^131^.

## Conclusions and perspectives

Because of special immune tolerance, universal immune cell therapies break the HLA mismatch barrier and reduce GVHD risks. Universal immune cell therapies have become strongly attractive, with improved availability and reduced costs compared to customized CAR-T-cell therapy.

The future of immune cell therapy does not include an alternation of CAR-T-cell therapy. The efficacy of all kinds of universal cell therapies must be improved, including expansion and persistence. The above-mentioned methods are combined to resolve these problems; however, the proliferation ability and persistence efficacy of universal immune cells are still insufficient. The combination of activated cytokines, membrane cytokines, anti-CD52 antibodies, and PEBLs may improve the efficacy of universal immune cells.

**Figure 3:**
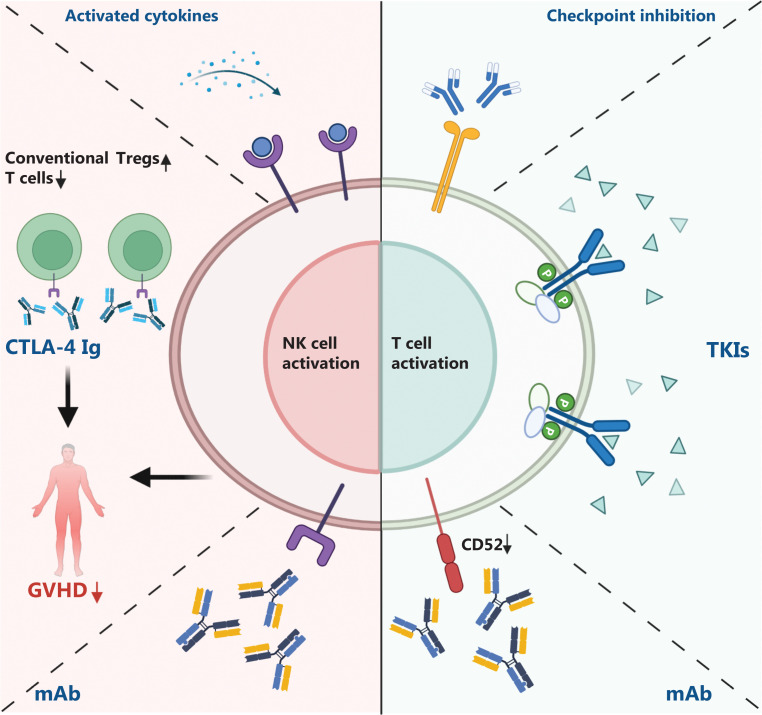
Potential combination of target drugs and universal immune cells. Combination strategies for NK and T cells currently differ. Activated cytokines stimulate NK cells *in vivo*, such that NK cells have high expansion ability. Antibodies are used to downregulate inhibitory signals to activate universal immune cells. Checkpoint inhibitors and TKIs play similar roles by different mechanisms. CTLA-4 Ig inhibits conventional T-cell activity and upregulates Tregs to downregulate GVHD risk. mAb, monoclonal antibody; TKI, tyrosine kinase inhibitors. The figure was created with BioRender (BioRender.com).

The trend in combination therapy of universal immune cell therapy is linkage with monoclonal antibodies^132^ (**Figure 3**). For R/R CD20-positive malignant lymphoma, seven of nine patients treated with *ex vivo*-expanded auto-NK cells combined with rituximab achieved a CR, with a median duration of 44 months^15^. Furthermore, with anti-CD52 monoclonal antibody (mAb) preconditioning, after infusion of anti-BCMA CAR-NK cells, 3 of 5 patients with refractory/relapsed (R/R) MM in the high-dosage group at least achieved very good partial remission (GVPR). The limitation of proliferation efficacy and the management of side effects with the use of precondition drugs and stimulators need further exploration. Compared with CAR-NK cell therapy, universal CAR-T-cell therapy has more obstacles. Anti-CD52 monoclonal antibody has been shown to be efficient at improving the persistence of allogenic CAR-T cells^28^. CD52 and cytoablative drugs, such as melphalan, have been applied in universal CAR-T-cell pre-treatment, and promising results of lowering the tumor burden has been achieved. Moreover, tyrosine kinase inhibitors (TKIs) are believed to be involved in the development of tumors and regulate the TME. TKIs and other immune modulators warrant further study. CD28 is a well-known T-cell co-stimulation molecule, and CD28 blockade of CTLA-4 has been used for GVHD prevention. The T-cell co-stimulation blockade agent, abatacept (CTLA-4 Ig), significantly decreases GVHD severity^133^, probably by inhibiting conventional T-cell activation, promoting Treg function, and simultaneously augmenting the anti-leukemia effects of NK cells^134–137^. Abatacept-primed DLIs after haplo-identical transplantation have been used to treat advanced hematologic malignancies. Only 3 of 12 patients with refractory aggressive B-cell lymphoma receiving abatacept-primed DLIs had disease progression 100 days post-transplant, and no patients had aGVHD^138^. In addition, no GVHD was reported in patients with refractory myeloma. The CD28-CD86 pathway may be the target of abatacept in myeloma cells, which demonstrated that abatacept-primed DLI is possible as a novel approach for myeloma treatment^139^. Moreover, compared to the conventional DLI group, the abatacept-primed DLI group had a lower GVHD and progression-free survival^140^. Thus, CTLA-4 Ig may be a good drug to combine with universal immune cell therapies to efficiently guarantee extremely low GVHD occurrence.
